# A Brace-Integrated Fiber Bragg Grating Sensor with a Multi-Hardness Silicone Strain Transfer Substrate for Knee Flexion Monitoring: A Proof-of-Concept Study

**DOI:** 10.3390/s26216939

**Published:** 2026-11-02

**Authors:** Seongmun Jeong, Sangwon Park, Wontae Choe, Chang Hyun Park, Dahun Jung, Jeesu Kim, Hyung-Hoi Kim, Chang-Seok Kim, Pilwon Hur, Hwidon Lee

**Affiliations:** 1 Department of Cogno-Mechatronics Engineering, Pusan National University, Busan 46241, Republic of Korea; smjeong99@pusan.ac.kr (S.J.); sangaone@pusan.ac.kr (S.P.); ckdgus9880@pusan.ac.kr (C.H.P.); jdh1585@pusan.ac.kr (D.J.); jeesukim@pusan.ac.kr (J.K.); ckim@pusan.ac.kr (C.-S.K.); 2 Engineering Research Center for Color-Modulated Extra-Sensory Perception Technology, Pusan National University, Busan 46241, Republic of Korea; 3 ARK Inc., Busan 46241, Republic of Korea; hhkim@pusan.ac.kr; 4 The Crystal Bank Research Institute, Pusan National University, Busan 46241, Republic of Korea; 5 Department of Mechanical and Robotics Engineering, Gwangju Institute of Science and Technology, Gwangju 61005, Republic of Korea; pilwonhur@gist.ac.kr

**Keywords:** fiber Bragg grating sensor, knee flexion monitoring, wearable sensor, knee brace, silicone strain transfer substrate, gait analysis

## Abstract

Continuous knee flexion monitoring is important for rehabilitation and gait analysis, but optical motion capture is generally confined to laboratories, and wearable electrical sensors may be affected by drift, misalignment, or electromagnetic interference. This study presents a brace-integrated system based on a fiber Bragg grating (FBG) embedded in a functionally segmented, multi-hardness silicone strain transfer substrate. The substrate was placed in a dedicated brace recess, avoiding a separate rigid transduction mechanism. A portable interrogation unit enabled wireless transmission and real-time brace-derived angle estimation during sit-to-stand and walking tasks. Calibration against brace hinge angles from 0° to 90° showed a nonlinear Bragg wavelength–angle relationship, modeled using a fourth-order polynomial whose order was selected using a separately acquired validation set that was also used for performance assessment. Validation based on 10 angle-wise mean estimates yielded R^2^ = 0.9992, RMSE = 0.90°, and MAE = 0.68°, with Bland–Altman 95% limits of agreement of −1.46° to +2.05°. The 0° stability record had a wavelength standard deviation of 1.41 pm and a linear trend of −0.193 pm/min, and Allan deviation analysis gave a wavelength noise floor of 0.089 pm at an averaging time of 24 s. Noise-equivalent angular variation ranged from 0.08° to 0.52°. During a sustained 30° hold, the estimated angle drifted at −0.089°/min, but comparison of the 0° and 30° records in wavelength units showed that the undeformed condition reproduced approximately 59% of that drift, although the non-simultaneous comparison does not establish a common-mode thermal contribution. An uncompensated temperature change of 1 K corresponds to between 0.31° and 1.98° of apparent angle change. Proof-of-concept measurements in two participants, both of whom are authors, produced brace-derived profiles qualitatively consistent with expected task kinematics but revealed a cumulative zero-offset shift of 3.2° across two sit-to-stand cycles. Because no simultaneous anatomical reference was acquired, the dynamic results establish feasibility and waveform plausibility rather than measurement accuracy. The results support the engineering feasibility of this brace-integrated optical architecture, while identifying temperature compensation and cyclic loading characterization as prerequisites for anatomical and clinical validation.

## 1. Introduction

Knee flexion and extension are fundamental components of lower-limb movement, and measurement of the knee flexion angle provides quantitative information on knee range of motion (ROM) during functional activities. Knee flexion angle measurement is therefore widely used in rehabilitation and clinical assessment. In patients with knee osteoarthritis, limitations in ROM can reflect functional status, whereas recovery of ROM following anterior cruciate ligament reconstruction can serve as an indicator of rehabilitation progress [1,2]. In particular, post-stroke stiff-knee gait, which has been reported in approximately 25–75% of patients with gait impairment, is characterized by reduced peak knee flexion during the swing phase [3,4,5]. Knee ROM during gait has also been reported to decrease significantly with advancing age [6,7]. In sports injury rehabilitation, repeated assessment of knee motion is required to evaluate functional recovery and support return-to-sport decisions [8,9]. Collectively, these applications highlight the need for quantitative assessment of knee flexion and extension beyond discrete measurements obtained during clinical visits. Accordingly, there is increasing demand for technologies capable of continuously and objectively measuring knee flexion angle during daily activities, including walking and sit-to-stand tasks. This demand has driven growing interest in wearable sensing technologies that enable real-time monitoring of knee flexion angle outside laboratory settings [10,11,12].

Optical motion capture systems, which are widely regarded as the gold standard for knee joint angle measurement, provide high measurement accuracy but generally require multiple cameras, marker placement, and dedicated measurement spaces, thereby restricting their use largely to laboratory settings [13,14]. Consequently, continuous knee flexion angle monitoring in clinical and daily-life settings remains difficult [15]. Various wearable alternatives have been proposed to overcome these limitations [16]; however, each sensing modality has characteristic sources of measurement error. Inertial measurement unit (IMU)-based systems estimate segment orientation and derive joint angles from accelerometer and gyroscope measurements, often supplemented by magnetometer data. However, these systems remain susceptible to integration drift and, when magnetometers are used, errors caused by magnetic disturbances [17,18]. Electrogoniometers are not affected by cumulative integration drift; however, because the instantaneous axis of rotation of the knee changes throughout flexion and extension, measurement errors can arise from sensor-to-joint-axis misalignment and sensor slippage during movement [19,20,21]. These limitations motivate the development of compact wearable sensing technologies capable of monitoring knee motion outside laboratory environments while minimizing modality-specific measurement errors.

Fiber Bragg grating (FBG) sensors are passive optical sensing elements that are immune to electromagnetic interference (EMI), can be implemented in compact and lightweight configurations, and allow multiple sensing points to be multiplexed along a single optical fiber [22,23]. These characteristics reduce the environmental constraints associated with electrical sensing modalities and make FBG sensors attractive for wearable knee flexion angle measurement. Existing FBG-based approaches can be broadly classified into systems that employ separate mechanical transduction structures to convert joint rotation into FBG strain and systems that incorporate FBG sensors into flexible substrates or textile structures. In the former, rigid mechanisms such as cantilevers, cams, and crank-slider mechanisms convert joint rotation into strain applied to the FBG sensor [24,25,26]. However, the use of additional mechanical transduction components increases structural complexity and hinders compact integration into wearable knee braces. In contrast, FBGs have been embedded in flexible silicone structures, incorporated into wearable belts or additively manufactured substrates, and attached to textile or polymeric supports for joint motion monitoring [27,28,29,30]. An instrumented knee brace incorporating a polymer-encapsulated FBG has also been demonstrated under static and walking conditions, while more recent studies have investigated hybrid photonic–inertial wireless systems and alternative optical demodulation schemes for knee motion monitoring [31,32,33]. These studies establish the feasibility of FBG-based wearable joint sensing; however, comparatively little attention has been given to the mechanical strain transfer architecture used to integrate the FBG into the brace. In particular, a brace-integrated substrate that assigns fixation, deformation accommodation, and FBG encapsulation to regions with different mechanical compliances remains insufficiently investigated.

In this study, we propose a brace-integrated knee flexion monitoring system based on an FBG embedded in a functionally segmented, multi-hardness silicone strain transfer substrate. The substrate comprises mechanically distinct regions designed to secure the sensor within the brace, accommodate deformation during brace rotation, and transfer axial strain to the protected FBG sensing region. The completed substrate is positioned within a dedicated recess formed directly in the brace, avoiding the need for a separate FBG-specific rigid transduction component, such as a cantilever, cam, or crank-slider mechanism. A portable interrogation unit with wireless data transmission was also developed to enable real-time brace-derived angle estimation during walking and sit-to-stand tasks without a tethered connection to an external acquisition system. The objective of this study is therefore not to establish anatomical knee angle accuracy, but to design and quantitatively characterize a brace-integrated strain transfer architecture at the brace level and to identify the error sources that must be addressed before anatomical validation is attempted. The proposed system was evaluated through short-term wavelength stability characterization, static calibration against reference brace hinge angles, separately acquired validation measurements, repeatability assessment, sustained-angle drift characterization, and dynamic measurements during walking and sit-to-stand tasks. The results demonstrate the feasibility of the proposed integration approach and indicate its potential for real-time knee flexion angle monitoring during functional movements, while further validation against an anatomical knee angle reference remains necessary for clinical application.

The contribution of this work is therefore threefold. The strain transfer medium itself acts as the transduction element, removing the separate rigid cantilever, cam, or crank-slider used in previously reported FBG knee angle devices. The medium is functionally segmented rather than uniform, so that fixation, deformation accommodation, and strain transfer are assigned to regions of different hardness instead of being carried by a single encapsulation material. Finally, the architecture is characterized quantitatively, through interval sensitivity, the Allan deviation noise floor, an exploratory comparison of drift under unloaded and loaded static conditions, and an explicit thermal error budget, rather than demonstrated qualitatively.

## 2. FBG Sensing Principle and Brace Angle Transduction Mechanism

An FBG is a wavelength-selective reflector formed by a periodic modulation of the refractive index along the core of an optical fiber. When broadband light propagates through the grating, a narrow spectral band satisfying the Bragg condition is reflected, while the remaining spectrum is transmitted. The reflected wavelength, referred to as the Bragg wavelength, is given by

λ_B_ = 2n_eff_Λ, (1)

where *λ*_B_ is the Bragg wavelength, *n*_eff_ is the effective refractive index of the fiber core, and Λ is the grating period.

Because the Bragg wavelength depends directly on *n*_eff_ and Λ, an FBG is sensitive to external measurands that alter either parameter. When the grating is subjected to uniform axial strain or a temperature change, both *n*_eff_ and Λ are altered, resulting in a shift in the reflected Bragg wavelength. For small changes, the normalized Bragg wavelength shift can be expressed as

(2) $$\frac{{\Delta \lambda }_{B}}{\lambda_{B}}=\left(1-p_{e}\right)\varepsilon +\left(\alpha +\xi \right)\Delta T,$$

where ε denotes the axial strain applied to the grating, p_e_ is the effective photoelastic coefficient, α is the thermal expansion coefficient of the optical fiber, ξ is the thermo-optic coefficient, and ΔT is the temperature change.

**Figure 1:**
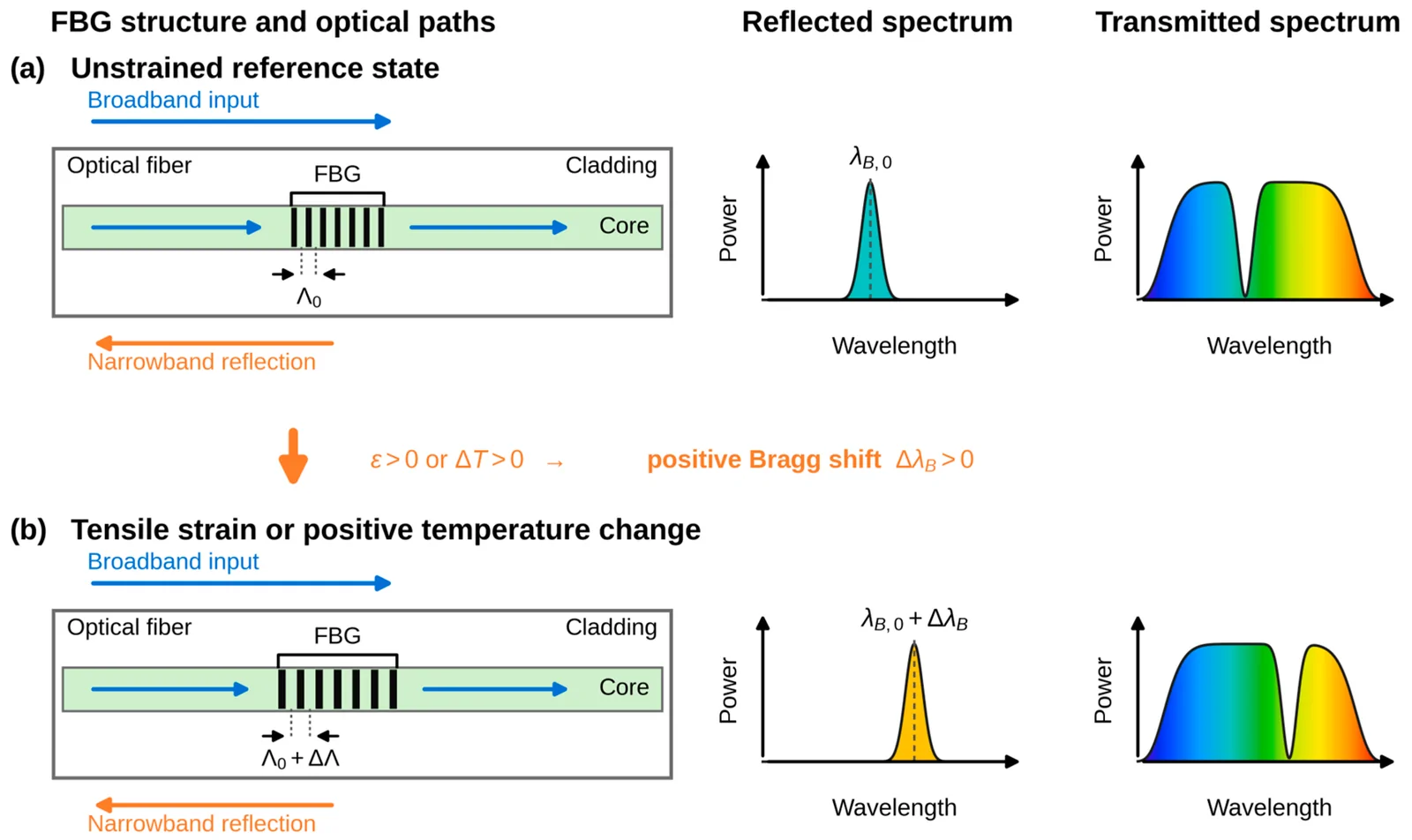
The operating principle of an FBG sensor. (**a**) In the unstrained reference state, the grating reflects a narrow band at the initial Bragg wavelength and produces a corresponding notch in the transmitted spectrum. (**b**) Tensile strain or a positive temperature change perturbs the grating period and effective refractive index, shifting the reflected band and transmission notch by Δλ_B_.

Under tensile strain, the grating period Λ increases and shifts the Bragg wavelength toward longer wavelengths. The strain-induced photoelastic effect decreases *n*_eff_ and partially offsets the contribution from the increased grating period. The net strain response is represented by (1 − p_e_)ε; because 1 − p_e_ > 0 for standard silica optical fibers, increasing tensile strain produces a positive Bragg wavelength shift. An increase in temperature also shifts the Bragg wavelength through thermal expansion and the thermo-optic response. Figure 1 compares the unstrained reference state with the red-shifted reflected and transmitted spectra produced by tensile strain or a positive temperature change.

In the proposed system, rotation of the brace hinge through an angle θ produces longitudinal deformation of the segmented silicone substrate. This deformation is transferred to the embedded FBG as axial strain, resulting in a Bragg wavelength shift according to Equation (2). Because both the deformation geometry and the effective strain transfer through the silicone regions vary with brace angle, the relationship between θ and Δλ_B_ is not assumed to be linear. Instead, the brace angle is estimated using an empirically determined calibration function,

(3) $$\hat{\theta }=f\left({\Delta \lambda }_{B}\right),$$

where $\hat{\theta }$ is the estimated brace angle and f is the calibration function. Accordingly, the calibration characterizes the combined response of the brace hinge, silicone strain transfer substrate, FBG, and interrogation system rather than the intrinsic response of the bare FBG alone.

For a strip anchored at a perpendicular offset h from the hinge axis, the change in anchor separation varies approximately as h·θ, that is, linearly with brace angle. The measured response was strongly superlinear, with the interval sensitivity increasing from 2.86 pm/° over 0–30° to 12.21 pm/° over 60–90°. This deviation from the geometric prediction is attributed to the mechanical behavior of the substrate: at small brace angles, the compliant Ecoflex 00-10 region takes up slack that remains because the fiber was installed without intentional pre-strain, and at larger angles the hyperelastic stiffening of that region increases the fraction of the imposed elongation that reaches the sensing region. The measured response was well described by an exponential stiffening form Δλ_B_ = A[exp(bθ/90) − 1]/[exp(b) − 1] with A = 0.589 nm and b = 2.23 (R^2^ = 0.996). Because this behavior is governed by the combined response of the hinge, substrate, grating, and interrogator, the brace angle is estimated using an empirically determined calibration function.

The walking and sit-to-stand experiments were short and were performed under nominally stable indoor temperature conditions. However, temperature was not measured independently, and the thermal term in Equation (2) is not negligible at the sensitivities achieved here: at 829.75 nm the thermal responsivity of a bare grating is approximately 5.4 pm/K, which corresponds to an apparent angle change of about 2.0° near full extension. Residual thermal contributions to the observed wavelength shift therefore cannot be excluded, and they are quantified explicitly in Section 4.1.

## 3. Materials and Methods

### 3.1. Wearable FBG-Integrated Knee Brace and Data Acquisition System

**Figure 2:**
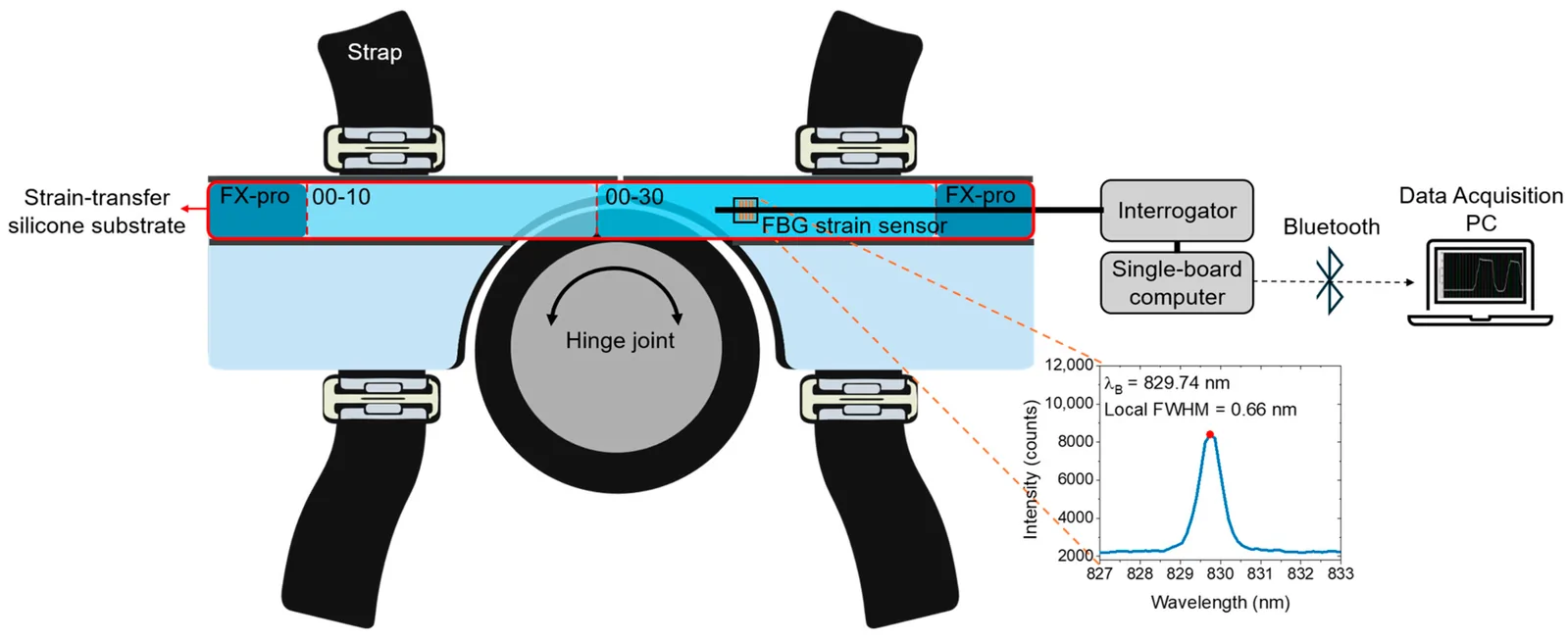
The architecture of the brace-integrated sensing system, including the segmented silicone strain transfer substrate, embedded FBG, interrogator, single-board computer, Bluetooth link, and data acquisition computer. Inset: measured local reflection spectrum with a peak at 829.74 nm and a local full width at half maximum of 0.66 nm.

Figure 2 illustrates the brace-integrated FBG sensing architecture and the associated data acquisition chain. The brace consists of two rigid segments secured to the thigh and shank by straps and connected by a hinge joint, allowing the brace to rotate with knee flexion and extension. A dedicated longitudinal recess accommodates the FBG-embedded silicone substrate, and the center of the FBG sensing region is positioned 60 mm from the hinge axis. Brace rotation elongates the substrate and transfers tensile strain to the FBG. The inset shows the local reflection spectrum associated with the grating used in the brace.

The strain sensor was a commercially inscribed fiber Bragg grating written in acrylate-coated SM800 single-mode fiber (FiSens GmbH, Braunschweig, Germany; specification sheet OC-0547-P2_14). The gratings were inscribed by the manufacturer using a femtosecond laser; the grating length was approximately 1 mm and the reflectivity exceeded 20%. The fiber as supplied carried two gratings, at manufacturer-specified center wavelengths of 819.927 nm and 829.930 nm. The 819.927 nm grating was damaged during assembly and the corresponding fiber section was removed, so the completed sensor contained the single grating at 829.930 nm, for which the manufacturer specified a full width at half maximum of 0.516 nm and a side-lobe suppression of −19.5 dB. A reflection spectrum recorded before assembly with an optical spectrum analyzer (AQ6370, Yokogawa Test & Measurement Corporation, Hachioji, Japan) gave an unstrained peak at 829.74 nm with a measured width of 0.66 nm; the broadening relative to the manufacturer value is consistent with the 0.12 nm spectral sampling interval of that instrument. In the completed system the reflected signal was acquired with an FBG interrogator module (FiSpec FBG X100, FiSens GmbH, Braunschweig, Germany), which has an operating wavelength range of 808–880 nm and selectable sampling rates of 1–200 Hz. The module extracted the peak Bragg wavelength and transferred the data through USB to a single-board computer (Raspberry Pi 4 Model B, Raspberry Pi Ltd., Cambridge, UK).

The interrogator, single-board computer, and battery were integrated in a portable enclosure. The single-board computer transmitted the wavelength data by Bluetooth to a data acquisition computer, where the calibration function converted the wavelength shift into a brace-angle estimate in real time. The wireless link eliminated the tether between the waist-mounted interrogation unit and the data acquisition computer during the functional tasks.

### 3.2. Design and Fabrication of FBG-Embedded Strain Transfer Silicone Substrate

**Figure 3:**
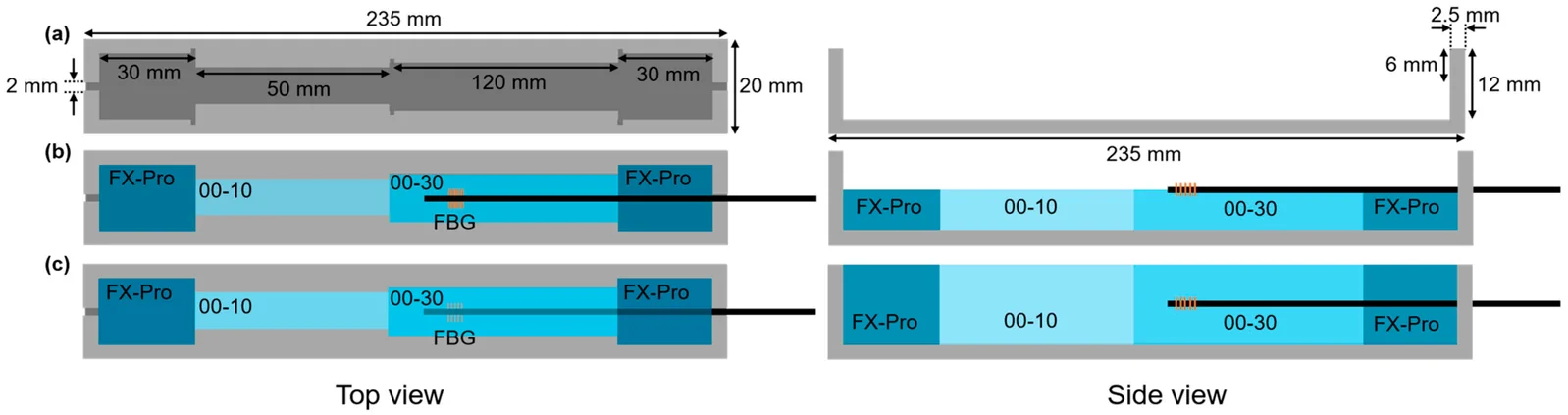
Design and sequential casting of the segmented silicone strain transfer substrate. (**a**) Mold geometry and section dimensions; (**b**) lower-layer casting and FBG placement; (**c**) upper-layer casting and complete FBG encapsulation. FX-Pro: Dragon Skin FX-Pro; 00-10: Ecoflex 00-10; 00-30: Ecoflex 00-30.

A custom mold was fabricated to produce the strain transfer substrate, as shown in Figure 3a. The mold had an overall length of 235 mm, width of 20 mm, and depth of 12 mm, with 2.5 mm thick end walls. A 2 mm wide notch extended 6 mm downward from the top edge of one side wall to route the optical fiber near the substrate mid-plane. The internal casting region was divided longitudinally into sections of 30, 50, 120, and 30 mm using removable partition walls.

Three silicone materials with different hardnesses were assigned distinct mechanical functions: Dragon Skin FX-Pro (Shore A2), Ecoflex 00-10 (Shore 00-10), and Ecoflex 00-30 (Shore 00-30) (Smooth-On Inc., Macungie, PA, USA). Dragon Skin FX-Pro was placed at both ends to secure the substrate within the brace recess and limit slippage. The highly compliant Ecoflex 00-10 region accommodated deformation associated with flexion and extension and transferred the resulting elongation to the adjacent Ecoflex 00-30 sensing region. Ecoflex 00-30 encapsulated the FBG, balancing strain transfer with mechanical protection of the grating.

The FBG was embedded using a two-stage casting process through the substrate thickness (Figure 3b,c). Before each step, Parts A and B of the corresponding silicone were mixed at a 1:1 mass ratio and vacuum-degassed to remove entrapped air. For the lower layer, Dragon Skin FX-Pro was cast into the two end regions and cured for 40 min. After the outer partition walls were removed, Ecoflex 00-10 was cast in its designated region and cured for 4 h. The central partition wall was then removed, and Ecoflex 00-30 was cast in the remaining region and cured for 4 h.

After the lower layer had cured, the acrylate coating was locally removed at designated fiber-fixation points outside the grating region. The bonding areas on the silicone were treated with a surface primer (LOCTITE SF 770, Henkel AG & Co. KGaA, Düsseldorf, Germany). The FBG was positioned without intentional pre-strain, and the locally uncoated fiber sections were bonded to the primer-treated surface. The upper half of the mold was then filled by repeating the same longitudinal sequence and material-specific curing times, fully encapsulating the FBG. Each silicone region was cured before the adjacent partition walls were removed, preserving the designed boundaries.

This process positioned the FBG near the mid-plane of the substrate thickness. In bending sensors, off-center placement is commonly used to increase curvature-induced axial strain [30]. Here, mid-plane placement was selected to reduce local bending strain and to favor the axial tensile strain produced by overall substrate elongation, while also reducing the risk of excessive strain and fiber fracture.

### 3.3. System Configuration, Calibration, and Measurement Protocols

**Figure 4:**
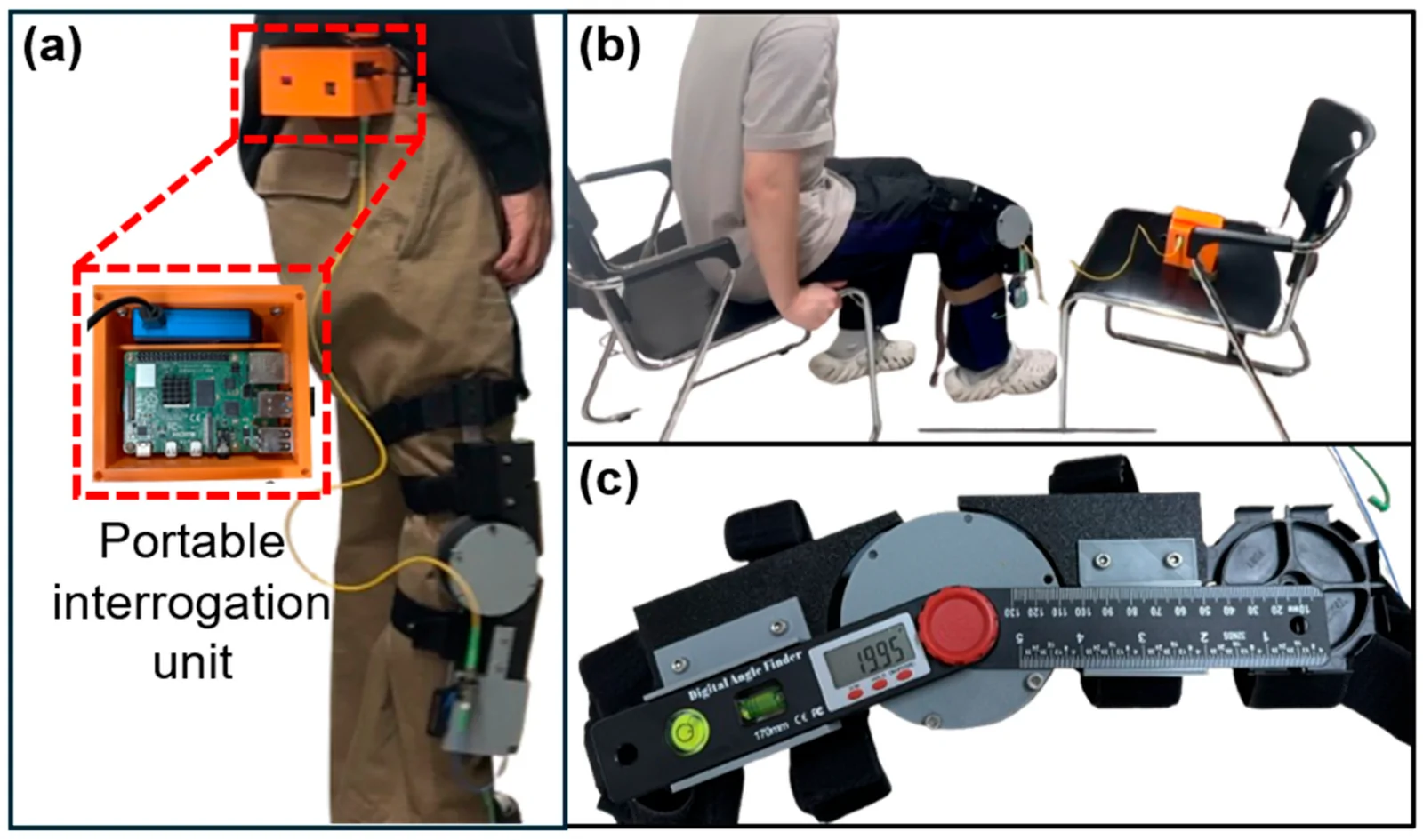
Experimental configurations. (**a**) Waist-mounted portable interrogation unit during walking; (**b**) sit-to-stand measurement; (**c**) static calibration with digital goniometer aligned to brace hinge axis.

Figure 4 shows the portable interrogation unit and the configurations used for walking, sit-to-stand, and static calibration. During dynamic testing, the unit was secured at each participant’s waist and connected by optical fiber to the FBG integrated in the brace. The Bragg wavelength was transmitted by Bluetooth to the data acquisition computer for real-time angle conversion.

Reference brace hinge angles were measured with a digital goniometer (BT170PB, BLUETEC, Daegu, Republic of Korea) mounted on the brace with its rotational axis aligned to the brace hinge. The stated accuracy of the goniometer was ±0.3°. For calibration, the brace was manually set from 0° to 90° in 10° increments and the corresponding Bragg wavelength shift was recorded. Because the response was nonlinear, a fourth-order polynomial was used,

(4) $$\hat{\theta }=a_{0}+a_{1}{\Delta \lambda }_{B}+a_{2}{\left({\Delta \lambda }_{B}\right)}^{2}+a_{3}{\left({\Delta \lambda }_{B}\right)}^{3}+a_{4}{\left({\Delta \lambda }_{B}\right)}^{4},$$

where a_0_–a_4_ are calibration coefficients and Δλ_B_ is the wavelength shift in nanometers relative to the acquisition baseline. Polynomial orders from two to five were fitted to the calibration data and compared on the separately acquired validation set; the fourth-order function gave the lowest validation RMSE and was adopted. Because that dataset was used both for order selection and for performance assessment, the reported errors should be interpreted as an internal held-out assessment rather than a fully independent test estimate. The fitted coefficient values (a_0_, a_1_, a_2_, a_3_, a_4_) were (−0.0907, 394.79, −796.07, 927.02, −467.61), with units of °, °/nm, °/nm^2^, °/nm^3^, and °/nm^4^, respectively. The coefficients are quoted to the precision required to reproduce the calibration curve to better than 0.01° over the calibrated range, which is well below the noise-equivalent angular variation. The empirical polynomial was restricted to the calibrated range of 0–90°; estimates outside this interval are extrapolations and were not clamped in the present implementation. The fitted function was then applied to a separately acquired validation dataset. The supplied validation export contained one angle-wise mean estimate at each of 10 reference angles.

Short-term wavelength stability was evaluated with the brace fixed at 0° using 8400 samples over 587.93 s. Time-dependent stability under sustained deformation was assessed with the brace fixed at 30° using 37,081 samples over 598.98 s. Linear drift was quantified as the ordinary least-squares slope over the full record; the first and last 60 s means were also compared for the sustained 30° hold. No additional filtering was applied in this reanalysis. Dynamic feasibility was evaluated in two participants during two complete sit-to-stand repetitions and during a walking trial comprising straight walking, a turn, and return walking. Peaks were identified using a minimum prominence of 20° and minimum separation of 5 s for sit-to-stand and 0.8 s for walking. These dynamic trials did not include simultaneous optical motion capture or another anatomical knee angle reference.

## 4. Results and Discussion

### 4.1. Static Characterization, Calibration, and Validation

**Figure 5:**
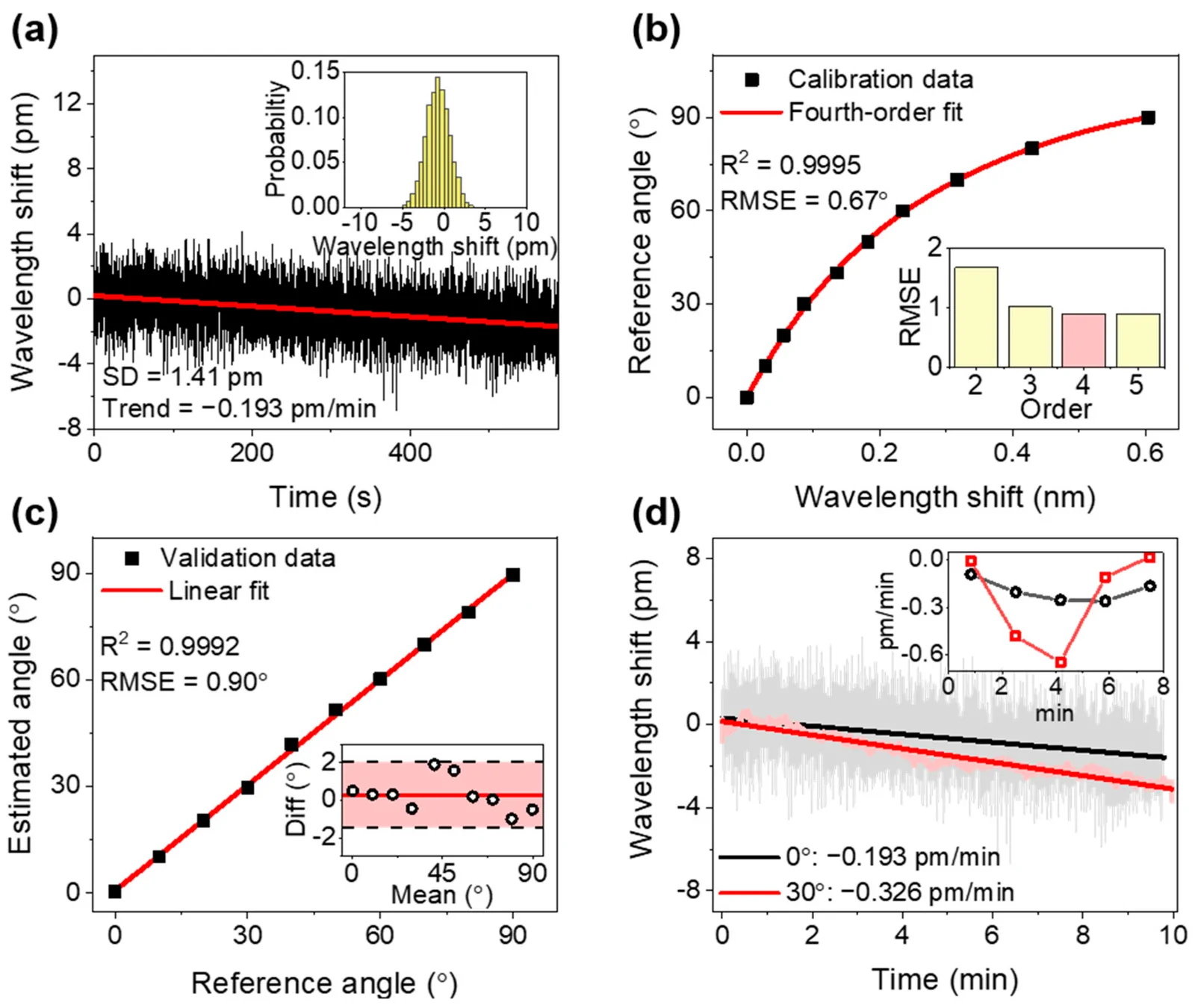
Static characterization, calibration, and validation after reanalysis of the supplied CSV exports. (**a**) Bragg wavelength shift at a reference brace angle of 0°, with the linear trend and shift histogram; (**b**) fourth-order polynomial calibration from 0° to 90°, with an inset comparing validation RMSE across polynomial orders; (**c**) validation using the exported angle-wise mean estimates, with the corresponding Bland–Altman plot as an inset; (**d**) wavelength-referenced comparison of the 30° and 0° records with their linear trends, and an inset showing the drift rate evaluated in successive 100 s blocks.

Throughout this section, the reference quantity is the brace hinge angle measured by the digital goniometer mounted on the hinge axis; the coefficients of determination and error metrics reported below therefore characterize the transduction chain from hinge rotation to Bragg wavelength, and should not be read as anatomical knee angle accuracy. Figure 5 summarizes the static characterization, calibration, and separately acquired validation results. With the brace fixed at 0°, the 587.93 s record had a mean Bragg wavelength shift of −0.746 pm and a sample standard deviation of 1.413 pm (Figure 5a). Ordinary least-squares regression identified a trend of −0.193 pm/min, and the mean of the last 30 s was 1.529 pm lower than that of the first 30 s. The detrended residual standard deviation was 1.304 pm. These results distinguish short-term wavelength fluctuation from the slower baseline change present in the same record.

**Figure 6:**
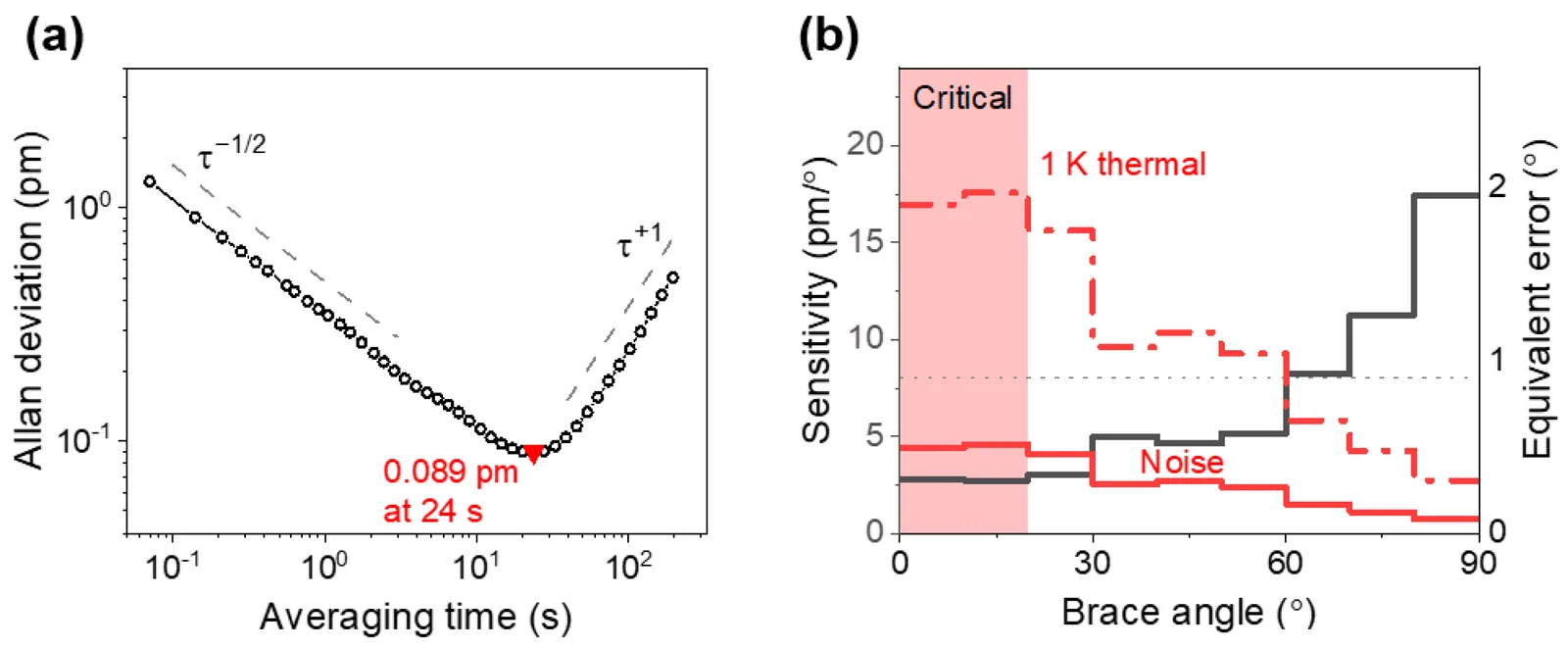
Noise characterization and error budget. (**a**) Overlapping Allan deviation of the 0° record, showing the white-noise region, the minimum at an averaging time of 24 s, and the drift-dominated region; (**b**) the interval sensitivity of the calibration curve and the corresponding noise-equivalent and thermally equivalent angle errors as a function of brace angle.

The noise structure of the same record was examined using the overlapping Allan deviation (Figure 6a). For averaging times below 2 s the Allan deviation followed a τ-1/2 dependence (fitted log-log slope −0.49), consistent with white noise in the wavelength readout. The Allan deviation reached a minimum of 0.089 pm at an averaging time of 24 s, which defines the best achievable wavelength resolution of the present system. For longer averaging times the Allan deviation increased with a slope of +0.95, indicating a near-deterministic drift term rather than random-walk noise. Averaging beyond approximately 24 s therefore degrades rather than improves the wavelength estimate, and long-duration monitoring requires drift compensation rather than longer integration.

The calibration curve in Figure 5b shows a nonlinear relation between wavelength shift and the reference brace angle. The fourth-order polynomial fit yielded R^2^ = 0.9995, a calibration RMSE of 0.67°, a mean absolute error of 0.51°, and a maximum absolute calibration residual of 1.29°. The total wavelength change between the exported 0° and 90° calibration points was 0.6038 nm, corresponding to a maximum axial strain of approximately 930 με, or 0.09%. Interval sensitivity ranged from 2.73 to 17.45 pm/° and increased overall with flexion, with a full-range mean of 6.71 pm/°. Because the calibration set contains only 10 points, the polynomial order was selected on the separately acquired validation set rather than by in-sample criteria: validation RMSE was 1.68° for second order, 1.02° for third order, 0.90° for fourth order, and 0.90° for fifth order; so, the fourth-order function was retained and no benefit was obtained from a higher order. This trend reflects the combined geometry and angle-dependent strain transfer of the brace-substrate assembly rather than a change in the intrinsic strain sensitivity of the FBG.

Using the 1.413 pm wavelength standard deviation and the interval sensitivities of the calibration curve, the noise-equivalent angular variation ranged from approximately 0.52° at low flexion to 0.08° at high flexion. This quantity maps wavelength variation through the local calibration response; it should not be interpreted as anatomical angular resolution or system accuracy.

Because the Bragg wavelength responds to temperature as well as strain (Equation (2)), the angular error associated with an uncompensated temperature change was estimated using the same interval sensitivities (Figure 6b). For a fiber Bragg grating at 829.75 nm, the thermal responsivity is approximately 5.4 pm/K, so a temperature change of 1 K corresponds to an apparent angle change of 1.98° over the least sensitive interval (10–20°), 1.08° over 30–40°, and 0.31° over 80–90°. The apparent error therefore exceeds the validation RMSE of 0.90° over most of the measurement range and is largest precisely where the mechanical sensitivity is lowest. Conversely, the 1.413 pm standard deviation observed over the 587.93 s stability record bounds the combined thermal and instrumental variation during that record to approximately 0.26 K of bare-grating-equivalent temperature change. Encapsulation in silicone, whose coefficient of thermal expansion exceeds that of silica by more than two orders of magnitude, is expected to modify, and may increase, the effective packaged thermal responsivity, so these values are taken as lower bounds. The magnitude of the thermal contribution also depends on the interval over which a measurement is referenced. Evaluating the Allan deviation of the 0° record (Figure 6a) at the durations of the dynamic trials gives 0.103 pm at 12.7 s and 0.096 pm at 34.1 s, equivalent to approximately 19 mK of bare-grating-equivalent temperature change; mapped through the lowest interval sensitivity of 2.73 pm/°, these correspond to approximately 0.04°, in line with the 0.040° obtained from the measured linear trend of the same record over 34 s. Because temperature was not recorded during the dynamic trials and the two sets of measurements were not acquired simultaneously, this figure characterizes short-term baseline instability under the static laboratory condition and should not be read as a direct upper bound on the thermal error during the dynamic trials. It nevertheless indicates that, under comparable conditions, the short-term baseline instability was small on the timescale of a single task, whereas slow drift becomes limiting for monitoring over minutes to hours.

As an illustrative post hoc sensitivity analysis, the trend measured in the 0° record was used as a surrogate baseline and subtracted from the trend of the 30° record, both expressed in wavelength. The residual is −0.133 pm/min against the original −0.326 pm/min, an illustrative reduction of 59%; at the local sensitivity of 3.69 pm/° at 30° the corresponding angular drift over ten minutes falls from 0.883° to 0.360°. This analysis does not demonstrate true common-mode compensation: the two records were acquired at different times, and the grating used as the surrogate baseline remained encapsulated in silicone and was therefore not mechanically isolated from the substrate. A simultaneous, strain-isolated but thermally co-located reference channel would be required to establish the suppression that is actually attainable. The 1.413 pm standard deviation of the 0° hold additionally bounds the combined thermal and instrumental variation during that record to approximately 0.26 K of bare-grating-equivalent temperature change; this constrains the combined variation under that static condition but is not a measurement of substrate temperature.

For the separately acquired validation data (Figure 5c), linear regression of the 10 angle-wise mean estimates against the reference angles yielded a slope of 0.9892, an intercept of 0.780°, and R^2^ = 0.9992. The RMSE, mean absolute error, and mean signed error were 0.90°, 0.68°, and 0.29°, respectively. Because the coefficient of determination quantifies association rather than agreement, a Bland–Altman analysis was also performed on the same 10 angle-wise means: the 95% limits of agreement were −1.46° to +2.05°, no proportional bias was detected (slope of the difference on the mean, −0.011°/°), and the mean difference did not differ significantly from zero (*p* = 0.33). Because these limits are based on means of repeated measurements rather than on individual observations, the corresponding interval for individual measurements was bounded by decomposing the observed variance into an angle-dependent systematic component and a within-angle random component. For any within-angle standard deviation up to 0.40°, the limits of agreement for individual observations would not exceed −1.60° to +2.18°, so the reported interval is insensitive to the unavailable replicate-level variability. The same decomposition gives a systematic standard deviation of at least 0.88°, which exceeds any plausible random component by more than a factor of two. Consistent with this, the maximum absolute error was 1.90° at 40°, followed by 1.59° at 50°, and these errors coincided with relatively large calibration residuals in the same range. To test whether the residual error is limited by the density of the calibration grid, the calibration function was refitted on subsets of the ten calibration points and evaluated on the same validation set. For each subset the polynomial order was re-selected on the same validation set subject to the number of points available, giving third order for the four- and five-point subsets and fourth order for the six-, seven- and ten-point subsets. The validation RMSE was 2.18° for a four-point grid at 30° spacing, 0.92° for five points, 0.93° for six, 0.89° for seven, and 0.90° for the full ten-point grid. Within the available dataset the validation RMSE therefore remained similar whenever five or more of the existing calibration points were retained, which suggests that calibration point density was not the dominant error source over the sampled range. Because no intermediate angles beyond the original ten-point grid were acquired, the possible benefit of a denser-than-ten-point calibration cannot be determined from the present data. The residual is more plausibly attributable to the accuracy of the goniometer reference, mechanical play in the brace hinge, and the uncharacterized loading–unloading hysteresis of the substrate. The stability of the selected polynomial order across the six-, seven-, and ten-point grids is consistent with a fit that is not dominated by model flexibility, although it does not by itself exclude overfitting, since the same validation set was used for order selection and for performance assessment.

With the brace held at 30° (Figure 5d), the full-record regression slope was −0.089°/min (R^2^ = 0.931), corresponding to a fitted change of −0.892° over 598.98 s. The first and last 60 s means were 29.595° and 28.795°, respectively, a difference of −0.800°. The raw standard deviation was 0.267°, whereas the standard deviation after linear detrending was 0.070°. To identify the origin of this drift, both static records were expressed in wavelength units and compared directly. The undeformed 0° record exhibited a trend of −0.193 pm/min against −0.326 pm/min at 30°, so approximately 59% of the sustained-deformation drift was reproduced in the absence of any sustained deformation. In addition, the 30° drift rate evaluated in successive 100 s blocks was near zero during the first 100 s, reached a maximum of 0.18°/min at approximately 4 min, and briefly reversed sign thereafter, whereas viscoelastic relaxation decays monotonically from the instant of loading. A stretched-exponential (Kohlrausch–Williams–Watts) fit to the 30° record returned an exponent of 1.48, outside the physically admissible range 0 < β ≤ 1 for relaxation processes. These observations indicate that the drift is not attributable to viscoelastic relaxation of the Ecoflex-based substrate alone [34]. The comparison suggests that a shared baseline component, largely independent of deformation and possibly thermal in origin through the (α + ξ) term of Equation (2), may contribute to the observed slow drift; because the two records were acquired non-simultaneously and temperature was not measured, it does not establish a common-mode thermal contribution, and the contributions cannot be separated from the present data. This is treated as a limitation.

### 4.2. Dynamic Measurements During Sit-to-Stand and Walking

**Figure 7:**
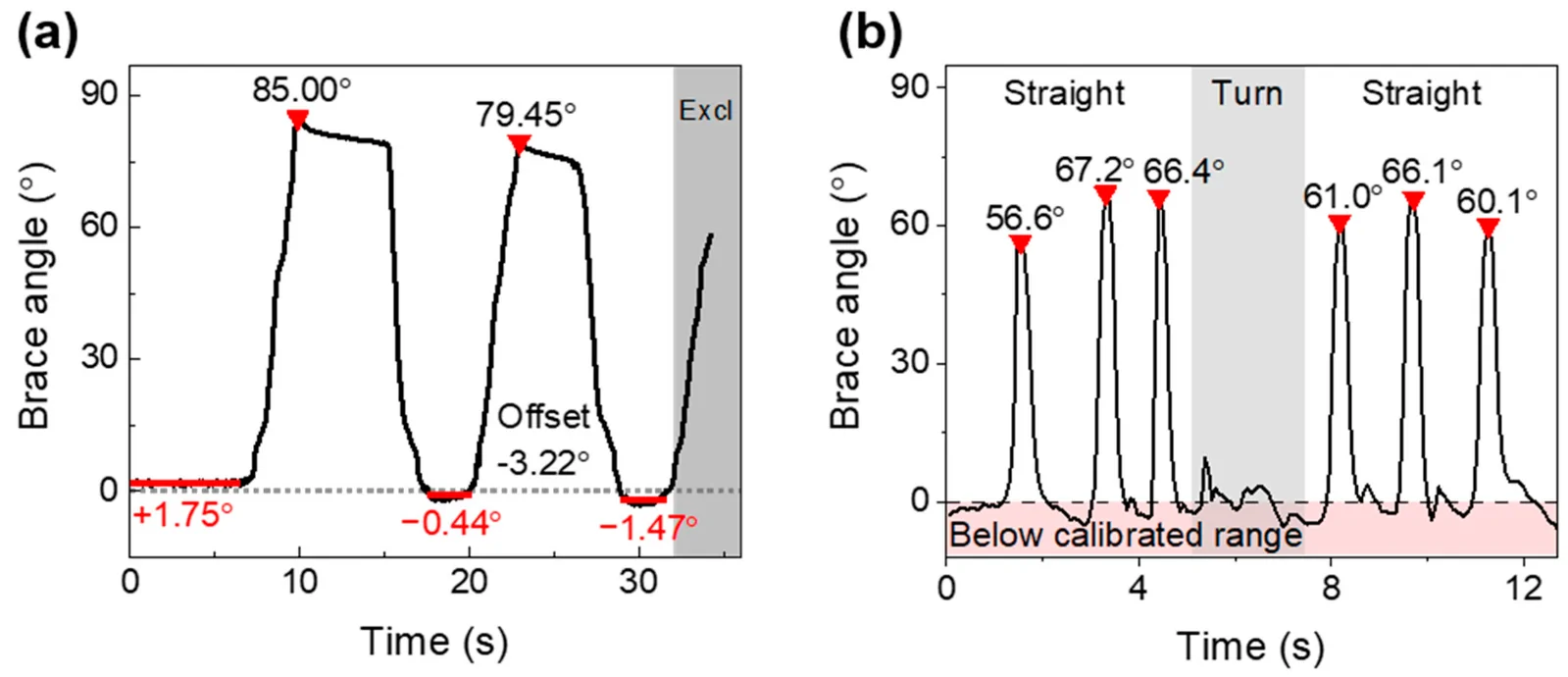
Brace-derived angle estimates during functional tasks. (**a**) Two complete sit-to-stand repetitions; the subsequent incomplete flexion after 32.2 s was excluded from the displayed analysis window. Red bars mark the mean of each standing interval and the cumulative zero-offset shift is annotated. Red triangles indicate the detected peaks. (**b**) A walking trial comprising straight walking, turning, and return straight walking, with the boundary of the calibrated range indicated. Peaks were detected with a minimum prominence of 20° and a minimum separation of 5 s for sit-to-stand and 0.8 s for walking.

Figure 7a shows the brace-derived angle estimate during two complete sit-to-stand repetitions. The estimated angle reached 85.00° in the first seated phase and 79.45° in the second. The 5.55° difference between seated peaks exceeded the static validation RMSE.

#### Cycle-Dependent Zero-Offset Shift

During the initial standing interval from 0 to 6 s the mean estimate was 1.75° with a sample standard deviation of 0.29°, whereas the standing intervals after the first and second repetitions gave means of −0.44° and −1.47°, that is, a cumulative zero-offset shift of −3.22° over 32 s. This rate, approximately 6.0°/min, exceeds the drift measured under sustained 30° deformation by nearly two orders of magnitude, indicating that repeated large-amplitude loading rather than sustained static loading governs baseline stability. Examining the internal trend of each standing interval is suggestive but not conclusive: the first two intervals fall at −1.31°/min and −5.13°/min, whereas the third rises at +3.20°/min, that is, toward the original baseline. These slopes are obtained from short and comparatively noisy 3.5 s windows and are consistent with, but do not establish, incomplete strain recovery having a time constant longer than the standing interval. The shift is also about two orders of magnitude larger than the short-term baseline instability of 0.04° measured over a comparable interval under static conditions, so it is unlikely to be explained by that instability alone, and the standing intervals become less repeatable as the sequence progresses, with sample standard deviations of 0.29°, 1.66°, and 1.77°. The shift is consistent with cyclic softening and incomplete strain recovery in the elastomeric substrate, and possibly with progressive settling of the substrate within the brace recess or with brace motion relative to the limb. For measurement reliability the consequence is that quantities derived within a single cycle, such as peak-to-peak range of motion, may be less sensitive to an additive offset than absolute angle referred to a single initial zero, although this was not validated here. Re-zeroing at a defined posture at the start of each trial would mitigate the accumulated offset, but it would not remove the underlying cyclic hysteresis and would not restore within-trial accuracy. The present observation rests on a single short window and cannot substitute for a dedicated loading–unloading protocol comprising multiple full cycles, recovery recorded for at least ten minutes after the final unloading, and residual offsets and recovery time constants reported per cycle. Such a protocol is required before the practical viability of the architecture for continuous monitoring can be established. Without a simultaneous dynamic reference, these changes cannot be assigned conclusively to posture variation, brace motion, mechanical hysteresis, or measurement error, and a dedicated loading–unloading hysteresis characterization is required. Knee flexion during sitting is influenced by seat height, posture, and anthropometry, and variability is reported even under standardized seating conditions [35].

In the walking trial (Figure 7b), three flexion peaks were observed in each straight-walking segment. The mean peak estimates were 63.4° (sample standard deviation = 5.9°) before turning and 62.4° (sample standard deviation = 3.2°) after turning, a difference of −1.0° for the return segment relative to the first. These magnitudes are qualitatively consistent with the approximately 60° peak knee flexion commonly reported during the swing phase of level walking [36]; the lower first peak, 56.6°, is consistent with gait initiation from standing. The stance-phase portions of the record extended below the calibrated range, reaching −6.6°, which is not anatomically attainable in a braced knee. These excursions reflect the combination of the zero-offset change described above and extrapolation of the calibration polynomial below Δλ_B_ = 0, and they illustrate that estimates outside 0–90° should be clamped or discarded in future implementations. This comparison supports waveform plausibility but does not constitute dynamic accuracy validation because the system output represented brace hinge angle and no concurrent anatomical reference was acquired.

At the measured static drift magnitude of 0.089°/min, the expected change over the 32.2 s sit-to-stand analysis window would be approximately 0.05°. This value is below the estimated noise-equivalent angular variation of 0.08–0.52° and suggests that sustained-load drift alone was unlikely to dominate these short recordings. The cycle-dependent zero-offset changes nevertheless indicate that repeated loading, mechanical hysteresis, brace movement, and longer monitoring sessions require separate evaluation.

### 4.3. Comparison with Previous FBG-Based Approaches and Study Limitations

In contrast to FBG knee angle devices that use a cantilever, cam, or crank-slider as a separate rigid transducer [24,25,26], the present system transfers brace rotation through a silicone substrate seated directly in the brace. Relative to previously reported flexible, textile, and polymer-embedded FBG approaches [27,28,29,30,31], its main design contribution is the functional segmentation of the strain transfer medium: the end regions provide fixation, the highly compliant region accommodates deformation, and the sensing region transfers strain while protecting the grating, so that three mechanically incompatible requirements are assigned to distinct regions rather than compromised within a single encapsulation medium. A further contribution is the quantitative characterization of what this architecture delivers and what it costs, through the interval sensitivity, the Allan deviation noise floor, an exploratory comparison of drift under unloaded and loaded static conditions, and the thermal error budget reported in Section 4.1. The portable interrogator and Bluetooth link further permit untethered acquisition. Recent photonic–inertial and spectrum demodulation studies illustrate complementary routes toward wireless or compact knee motion monitoring [32,33]. Because device geometries, reference standards, angle ranges, and test protocols differ across studies, the present results should not be interpreted as a direct performance superiority claim.

This study has several limitations. First, static calibration and validation used the brace hinge angle rather than the anatomical knee flexion angle; soft-tissue motion, brace slippage, and hinge-to-joint-axis misalignment were therefore not quantified, and the reported coefficient of determination and error metrics characterize the reproduction of hinge rotation only. Establishing anatomical accuracy requires concurrent optical motion capture or an equivalent validated reference, which was not available for the present measurements. Second, only two participants were included, both of whom are authors of this work, and dynamic trials lacked a simultaneous reference system, so the cohort is not representative and the measurements were not blinded. Third, temperature was not measured independently. The error-budget analysis indicates that an uncompensated change of 1 K would produce up to 2.0° of apparent angle change near full extension, and the comparison of the 0° and 30° records suggests that a shared baseline component may contribute to the observed slow drift, although the non-simultaneous acquisition does not establish a common-mode thermal contribution. An independent thermal characterization of the packaged sensor could not be performed in the present study because the measurement assembly had been disassembled; holding the brace at a fixed angle in a temperature-controlled chamber would yield the packaged thermal responsivity directly and appears readily achievable. Fourth, loading–unloading hysteresis was not characterized, and the cumulative zero-offset shift of 3.2° observed over two sit-to-stand cycles indicates that cyclic rather than sustained loading governs baseline stability [37]. Fifth, calibration used one device and 10 angle levels, and the fourth-order polynomial should not be extrapolated beyond 0–90°. Sixth, the fiber was installed without intentional pre-strain, which leaves a low-sensitivity region near full extension where the clinical information content of knee kinematics is often highest. Seventh, inter-device fabrication variability, donning repeatability, cyclic durability, and long-duration performance were not evaluated. Taken together with the cycle-dependent zero-offset shift reported in the Section Cycle-Dependent Zero-Offset Shift, these omissions mean that the practical viability of the present architecture for continuous daily monitoring is not established by the data reported here. Finally, the original acquisition rates, any resampling or filtering before export, and several participant–protocol details require confirmation before submission. Future studies should incorporate simultaneous motion capture or validated wearable references, larger and clinically relevant cohorts, multiple fabricated sensors, a strain-isolated but thermally co-located reference grating for temperature compensation, controlled pre-strain to improve low-angle sensitivity, and prolonged cyclic testing.

Three routes are available for suppressing the thermal term. Referencing each trial to a known posture immediately beforehand can reduce the influence of an initial slowly varying offset during short tasks, but it does not correct within-trial thermal changes or cyclic hysteresis. Measuring the packaged thermal responsivity and correcting for it would extend this to longer records; such a measurement could not be performed here because the assembly had been disassembled, but holding the brace at a fixed angle in a temperature-controlled chamber would provide it directly. For continuous monitoring, a second, strain-isolated but thermally co-located reference grating in the same fiber would allow the shared baseline term to be subtracted [38]. The interrogator has sufficient spectral range to accommodate an additional grating, so no change to the interrogation architecture would be required; implementing the approach would, however, require fabrication of a new dual-grating sensor in which one grating is mechanically decoupled from the substrate while remaining at the same temperature. This last route is also the most robust to the properties of the encapsulant. Because the coefficient of thermal expansion of the silicone exceeds that of silica by more than two orders of magnitude, thermal expansion of the substrate is transferred to the fiber as apparent strain and raises the packaged thermal responsivity above the bare-grating value of 5.4 pm/K used in the error budget, so the values reported there are lower bounds. A stiffer or lower-expansion encapsulant would reduce this cross-sensitivity, but would also reduce the strain transferred to the grating and remove the compliance that accommodates brace deformation and protects the fiber; the choice of medium is therefore a trade-off rather than a free parameter. Optical rejection of the shared baseline term addresses the thermal contribution without incurring that penalty and remains valid whatever the packaged responsivity proves to be.

## 5. Conclusions

This study developed a brace-integrated FBG knee flexion monitoring system based on a functionally segmented, multi-hardness silicone strain transfer substrate. The substrate directly couples brace rotation to the protected FBG without a separate rigid cantilever, cam, or crank-slider mechanism, while a portable interrogation unit provides wireless real-time readout. Static calibration over 0–90° produced a nonlinear wavelength–angle response described by a fourth-order polynomial, the order of which was selected on the separately acquired validation set. Validation based on 10 angle-wise mean estimates against the brace hinge reference yielded R^2^ = 0.9992, RMSE = 0.90°, and MAE = 0.68°, with Bland–Altman 95% limits of agreement of −1.46° to +2.05°. Allan deviation analysis gave a wavelength noise floor of 0.089 pm at 24 s averaging, and the noise-equivalent angular variation was 0.08–0.52°. The 0° wavelength record showed a trend of −0.193 pm/min and sustained deformation at 30° produced −0.326 pm/min; the non-simultaneous comparison suggests that a shared baseline component may contribute to the observed drift, but it does not establish a common-mode thermal contribution. An uncompensated temperature change of 1 K corresponds to 0.31–1.98° of apparent angle change.

Proof-of-concept sit-to-stand and walking measurements generated real-time brace-derived profiles that were qualitatively consistent with expected task kinematics, while revealing a cumulative zero-offset shift of 3.2° across two sit-to-stand cycles that is not predicted by the static drift figure. These measurements were obtained from two participants, both of whom are authors, without blinding and without a simultaneous anatomical reference; they establish engineering feasibility at the brace level and do not support any claim of measurement accuracy, nor do they generalize to clinical populations. Direct characterization of thermal responsivity and loading–unloading hysteresis, simultaneous anatomical reference measurements, larger cohorts, repeatability across fabricated sensors and repeated donning, and long-term durability testing are required before clinical or home-rehabilitation use can be established.

## Data Availability

The data supporting the findings of this study are available from the corresponding author upon reasonable request, subject to participant privacy and institutional requirements.
